# Assessing the impact of the introduction of an electronic hospital discharge system on the completeness and timeliness of discharge communication: a before and after study

**DOI:** 10.1186/s12913-017-2579-3

**Published:** 2017-09-05

**Authors:** Rajnikant L. Mehta, Bryn Baxendale, Katie Roth, Victoria Caswell, Ivan Le Jeune, Jack Hawkins, Haya Zedan, Anthony J. Avery

**Affiliations:** 1 School of Medicine, University of Nottingham, Queens Medical Centre, Nottingham, NG7 2UH England; 2 Nottingham University Hospitals NHS Trust, Queens Medical Centre, Nottingham, NG7 2UH England; 3 University Hospitals Leicester, Emergency Care Intensive Support Team, Leicester, England; 4 Department of Health Informatics, College of Health Sciences, Saudi Electronic University, Riyadh, Saudi Arabia

**Keywords:** Discharge summaries, Gold standard auditing tool, New electronic discharge system, Completeness

## Abstract

**Background:**

Hospital discharge summaries are a key communication tool ensuring continuity of care between primary and secondary care. Incomplete or untimely communication of information increases risk of hospital readmission and associated complications. The aim of this study was to evaluate whether the introduction of a new electronic discharge system (NewEDS) was associated with improvements in the completeness and timeliness of discharge information, in Nottingham University Hospitals NHS Trust, England.

**Methods:**

A before and after longitudinal study design was used. Data were collected using the gold standard auditing tool from the Royal College of Physicians (RCP). This tool contains a checklist of 57 items grouped into seven categories, 28 of which are classified as mandatory by RCP. Percentage completeness (out of the 28 mandatory items) was considered to be the primary outcome measure. Data from 773 patients discharged directly from the acute medical unit over eight-week long time periods (four before and four after the change to the NewEDS) from August 2010 to May 2012 were extracted and evaluated. Results were summarised by effect size on completeness before and after changeover to NewEDS respectively. The primary outcome variable was represented with percentage of completeness score and a non-parametric technique was used to compare pre-NewEDS and post-NewEDS scores.

**Results:**

The changeover to the NewEDS resulted in an increased completeness of discharge summaries from 60.7% to 75.0% (*p* < 0.001) and the proportion of summaries created under 24 h from discharge increased significantly from 78.0% to 93.0% (*p* < 0.001). Furthermore, five of the seven grouped checklist categories also showed significant improvements in levels of completeness (*p* < 0.001), although there were reduced levels of completeness for three items (p < 0.001).

**Conclusion:**

The introduction of a NewEDS was associated with a significant improvement in the completeness and timeliness of hospital discharge communication.

## Background

Hospital discharge summaries are a key communication tool for patient safety issues [1] as incomplete or untimely communication of information can lead to increased risk of hospital readmission and other associated complications. Admission to hospital often leads to new diagnoses, important investigation results and changes to treatment plans. Hence, it is very important that hospital discharge communications are accurate, complete and reach primary care teams in a timely manner [2–4].

Incomplete discharge summaries can have an adverse impact on patient safety [1], potentially causing increased avoidable hospital readmission, unnecessary complications and at the most extreme, patient mortality [4–6]. As the patient is particularly vulnerable during the period immediately after discharge, it is essential that discharge summaries are not only completed to a high standard but also that they are communicated to General Practitioners (GPs) in a timely manner. The lack of timeliness can also lead to an increased likelihood of unfavourable outcomes [2] and inappropriate utilization of health care resources. Re-admission is a major problem in hospitals, particularly in acute medical units where there is often a waiting list for beds and patients needing immediate medical attention. Re-admission of patients as a result of an incomplete discharge summary is avoidable and unnecessary [6, 7].

Hospital doctors sometimes view discharge summaries as a chore and can rush the completion of these communications, taking shortcuts which can lead to important information being overlooked and omitted either intentionally or otherwise [8]. The level of completion of each summary can vary and Grimes et al., found that one of the most common pieces of information being left off discharge summaries was ‘medication changes’, either what medications had actually been changed or the reason why [9, 10]. Changes to medication regimens is crucial information that must be forwarded to primary care. This is especially a concern in more complex patients with multiple morbidities who take multiple medications.

In 2008, the Royal College of Physicians (RCP) produced guidelines designed to improve the quality of discharge summaries and standardise their format for transmission between care sectors [11]. The template was designed to contain all the information necessary to correctly inform the GPs of the hospital episode and thus maintain continuity of care [11, 12].

In 2011, Nottingham University Hospitals NHS Trust (NUHT) implemented a new electronic discharge system (NewEDS) based on this gold standard template, having already moved away from traditional forms of writing discharge summaries (hand-written or dictated). The new system was developed ‘in house’ and is not commercially available. It was designed to incorporate the fields recommended in the RCP guidelines. Demographic information was auto-imported, but the hospital did not have an Electronic Health Record at the time and so clinical data and medications had to be manually recorded.

The new system replaced a single sheet of paper on which was written the patient’s details, diagnoses, medications and follow up arrangements. The NewEDS discharge summaries were transferred electronically to the relevant general practice computer systems using the NHS Spine, which provides a secure data connection.

Our objective was to evaluate whether the introduction of the NewEDS brought about improvements in the completeness and timeliness of discharge communication. In terms of ‘completeness’ we focused on the RCP mandatory clinical and administrative data.

## Methods

### Setting

We retrospectively studied patient discharge summaries records from two wards (named B3 and D57) which comprised part of the acute medical unit at NUHT in the UK. These wards were selected as they managed a full range of acute medical adult patients in a high-pressure environment with a high patient turnover. Ward B3 is focused on short-stay patients with an anticipated length of stay of less than 48 h. The average length of stay in B3 to direct discharge is 15 h. Ward D57 patients were more likely to require longer admissions. Most patients transferred from ward D57 to the base wards of other specialities leading to fewer direct discharges, however the patients discharged from this ward tended to be older and have more complex medical background.

### Selecting cases

The NewEDS was established on the 5th July 2011. Four (equally distanced) time periods were chosen before this date and four after to collect a sample of discharge summaries in order to address the aims of the study.

The periods examined were the weeks commencing 16th August 2010, 15th November 2010, 14th February 2011, 16th May 2011, 15th August 2011, 14th November 2011, 13th February 2012, and 14th May 2012. These dates were chosen as they did not cover any bank holidays or any other significant event that was felt might affect the level of completion and timeliness of the discharge summaries. Furthermore, one of the collection periods before and after NewEDS introduction was selected to include a week in mid-August close to the changeover period for new junior doctors to give a fair reflection of the potential effect of inexperience on the findings. Full weeks were looked at in order to assess a variety of inpatients as well as multiple doctors completing the summaries.

For each of the eight weeks in this study a random sample of 50 patients from each of the wards was created. Where there were fewer than 50 discharges, all were included. The list of patients contained basic demographic information (e.g. gender, date of birth), and admission and discharge dates and times were recorded from this source. Patient data were accessed using the Nottingham Information System (NotIS) database by two medical researchers (KR, VC), who received training in its use for purposes of data abstraction. Patients were identified on the system using their ‘K number’ (a unique number assigned to each patient treated at NUHT) and the discharge summary from the particular inpatient stay was then located.

### Recording the data

The two trained medical researchers inspected both the pre-NewEDS and post-NewEDS discharge summaries and recorded the data contained within them using a data collection tool that had previously been used in a PhD project [13]. This data collection tool was developed by HZ in 2009 in consultation with the RCP using their standards, summary headings and definitions. [11]

The data collection tool took the form of a checklist, with two columns for items to be recorded as present or not present; the checklist contained 57 items under 7 headings (GP Information, Patient Information, Admission Information, Discharge Information, Clinical Information, Advice Recommendations and Future Plan, and Person Completing Summary).

**Fig. 1:**
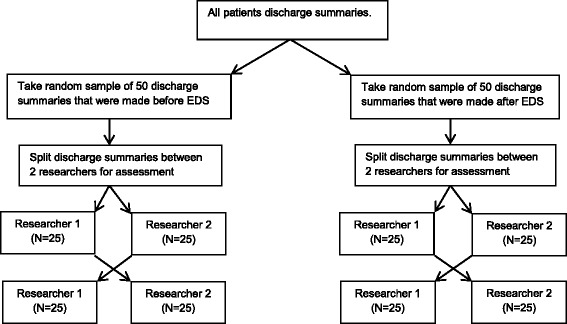
Flow chart depicting process and assessing agreement

To ensure the two medical researchers were assessing the data in the same way, agreement was analysed throughout the study. To assess agreement, discharge summaries sampled were crossed over and checked independently by the other medical researcher. This process was conducted for 50 discharge summaries before and after changeover respectively implying 100 summaries crossed over and re-analysed (Fig. 1). Agreement between the 2 medical researchers was found to be 99.23%. It was not possible to ‘blind’ the researchers as to which discharge summaries were completed before, and which were completed after, the introduction of the NewEDS.

### Inclusion and exclusion criteria

To be included in the study, patients had to have been discharged from hospital, from either ward, during the eight weeks examined. Patients were excluded if they were transferred to a different ward, had a missing discharge summary or if the researchers were not authorized to access the summary (this occurred if the summary contained sensitive information).

### Data analysis

There were two primary outcome variables. The first was the percentage completeness score of the discharge summaries in relation to 28 items of information considered mandatory by the RCP [11, 12]. The second was the percentage of discharge summaries created within 24 h of a hospital discharge [12].

The mean percentage completeness score before and after introduction of NewEDS was compared using an independent t-test. For categorical data, percentages were used and the chi-squared test was used to determine statistical significance. Where continuous variables like completeness and timeliness were not normally distributed non-parametric methods (Mann-Whitney U-test) were applied and the median with quartile ranges were presented. Statistical results pertaining to normally distributed continuous variables are presented with mean and standard deviation (SD), non-normally distributed variables with medians and quartile and categorical variables with percentages respectively. Data were analysed using STATA 13 and results were considered as statistically significant if *p* < 0.05 (two-sided). When pertinent, 95% confidence intervals (95% CI) were calculated.

### Sample size

A sample size calculation was conducted showing that, given a minimum 5% disagreement and assuming a 95% confidence interval width of 5%, a minimum of 292 discharge summaries needed to be analysed.

## Results

### Description of sample

During the study period 773 hospital discharge summaries were found to be eligible for inclusion. The discharge summaries were extracted from two wards (B3:400(51.7%) and D57:373(48.3%)) pre and post-NewEDS (pre-NewEDS:386(49.9%) and post-NewEDS:387(50.1%)) (Table 1). It can be seen in Table 1 that 50 were sampled for each time period for B3. There were fewer than 50 patients for some of the time periods for D57. Appendix 1 displays in more detail the number of discharge summaries excluded for each reason, per time point and ward. We found that the number of Total excluded discharge summaries decreased during Post-NewEDS compared to Pre-NewEDS period respectively.

**Table 1:** Discharge summaries examined for each time period, and by ward

Time-point	Ward				Total
	B3		D57		
	Number of records reviewed (%Target met)	Denominator from which records were selected	Number of records reviewed (% Target met)	Denominator from which records were selected	
Pre-NewEDS					
August 2010	50 (100)	179	50 (100)	61	386
November 2010	50 (100)	158	43 (86)	63	
February 2011	50 (100)	213	43 (86)	57	
May 2011	50 (100)	177	50 (100)	59	
Post-NewEDS					
August 2011	50 (100)	188	50 (100)	76	387
November 2011	50 (100)	215	48 (96)	51	
February 2012	50 (100)	127	47 (94)	52	
May 2012	50 (100)	214	42 (84)	47	

The recording of gender was present in all discharge summaries. The proportions of male and female were similar in each ward (B3(49.5% vs 50.5%) and D57(45.6% vs 54.4%); *p* = 0.275). However, patients age differed between the two wards and was significantly higher in D57 compared to B3 (mean(SD): 64.2(23.2) vs 57.2(21.2); *p* < 0.001).

### Completeness

**Fig. 2:**
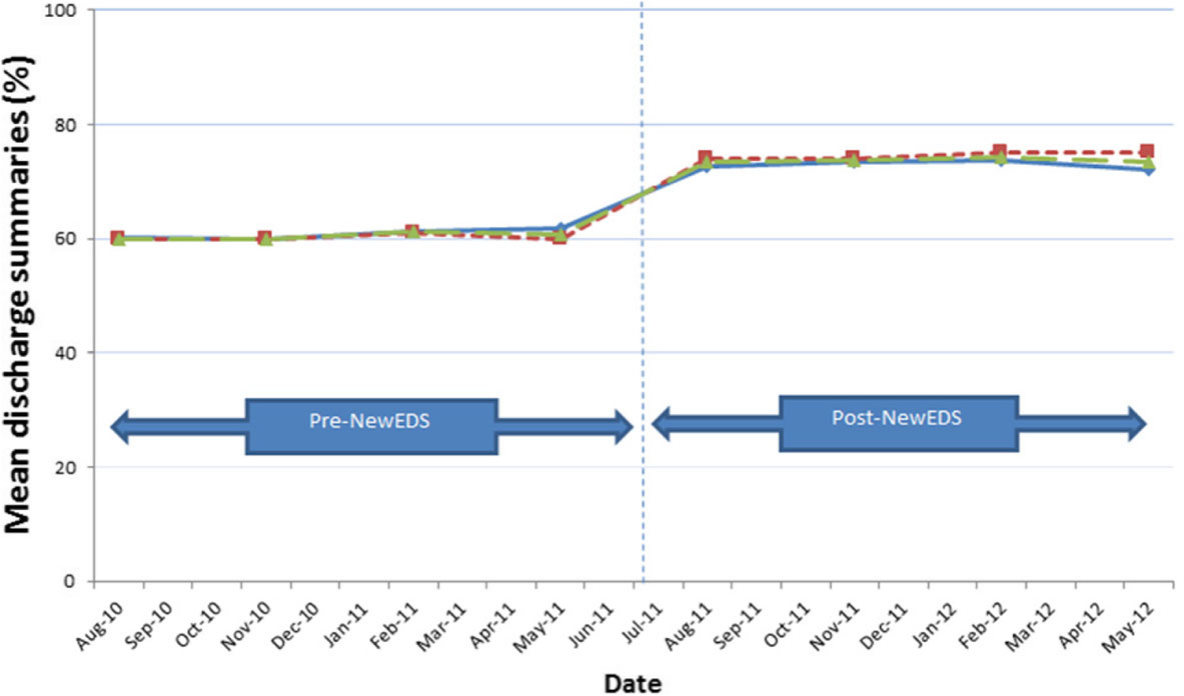
Mean percentage completeness scores of items considered mandatory by the Royal College of Physicians.  Ward B  Ward D57  Combined

The percentage completeness scores are illustrated in Fig. 2. The median percentage completeness score of RCP mandatory data pre and post-NewEDS was significantly higher post-NewEDS compared to Pre-NewEDS (Median (quartiles): 75.0%(75.0,75.0) vs 60.7%(57.1,64.3), p < 0.001(Mann-Whitney)). This demonstrates that completeness of RCP mandatory fields increased by 14.3% after implementation of the NewEDS.

### Timeliness

**Fig. 3:**
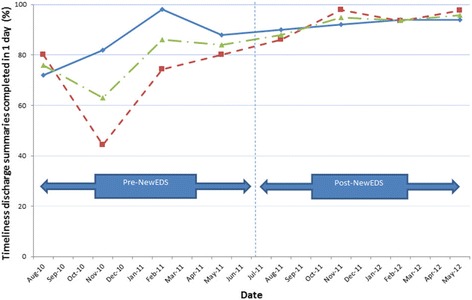
Discharge summaries created within 24 hours of a patient being discharged across the eight time periods.  Ward B  Ward D57  Combined

In relation to the timeliness of the discharge summaries score being completed, there was a significant decrease in days for completion after the introduction of the NewEDS (mean number of days: 4.2 vs 2.7, *p* < 0.001 (Mann Whitney)). Summarising the scores of both wards (B3 and D57) a significant increase was found in the post-NewEDS timeliness of discharge summaries completed within one day compared to pre-NewEDS (93.0% vs 78.0%, p < 0.001). Results pertaining to both wards also show that for the final four time-points, the proportion of timely discharge summaries were approximately 90% and above, reaching a peak of 97.6% in May 2012 (Fig. 3).

### RCP grouping and mandatory list

The RCP checklist had 56 items (signature was excluded) that could be marked as either ‘present’ or ‘not present’; these items were then grouped into seven categories: 1) GP details; 2) Patient details; 3) Admission details; 4) Discharge details; 5) Clinical information; 6) Advice, recommendations and future plan; 7) Person completing summary. For each category a tally of the number of items completed was calculated. Analyses were performed on each category and to assess whether there was a significant change in completeness between pre-NewEDS and post-NewEDS.

The completeness of categories 1–5 significantly increased after the change in EDS, while the completeness of categories 6 and 7 significantly decreased (Table 2).

**Table 2:** Results for the seven grouped Royal College of Physicians checklist categories before and after the implementation of the new electronic discharge system

	Number of items recorded		
Category(N: Number of items)	Before (Median (25th,75th quartiles))	After (Median (25th,75th quartiles))	*P* value (Mann Whitney Test)
1) GP Details (3)	2 (2,2)	3 (3,3)	<0.001
2) Patient Details (7)	4 (4,4)	6 (6,6)	<0.001
3) Admission Details (6)	3 (3,3)	4 (4,4)	<0.001
4) Discharge Details (8)	3 (3,3)	4 (4,5)	<0.001
5) Clinical Information (14)	6 (5,7)	8 (7,9)	<0.001
6) Advice, Recommendations and Future Plan (13)	3 (3,4)	3 (0,4)	<0.001
7) Person Completing Summary (5)	3 (2,3)	1 (1,1)	<0.001

Of the 56 RCP items, 28 items were RCP mandatory fields and 12 items showed highly statistically significant (*p* < 0.001) differences in completeness between the two systems: nine items showed improvements using the NewEDS and of these three were related to medication measures; three items showed decreased levels of completeness when using the NewEDS (Table 3). Table 3 also shows four non-mandatory items that demonstrated improvements when using the NewEDS and four items which showed decreased levels of completeness.

**Table 3:** Results of highly statistically significant chi square tests for completeness of 56 items on Royal College of Physicians checklist before and after change in electronic discharge system

	Category Number	Item name - Completeness	Before: N(%) *N* = 386	After: N(%) *N* = 387	P Value ^a^
Administrative: GP, Patient, Administration and Discharge details	1	GP Practice code ^c^	0 (0.0)	362 (93.5)	<0.001 ^b^
	2	Gender ^c^	1 (0.3)	374 (96.6)	<0.001 ^b^
	2	Patient Telephone Number ^c^	1 (0.3)	364 (94.1)	<0.001 ^b^
	3	Method of Admission	47 (12.2)	375 (96.9)	<0.001
	4	Date of Discharge ^c^	340 (88.1)	385 (99.5)	<0.001 ^b^
	4	Destination Address ^c^	0 (0.0)	373 (96.4)	<0.001 ^b^
	4	Discharging Consultant ^c^	356 (92.2)	387 (100.0)	<0.001 ^b^
Clinical: Clinical information	5	Diagnosis at Discharge ^c^	376 (97.4)	387 (100.0)	0.001 ^b^
	5	Medication Changes ^c^	304 (78.8)	386 (99.7)	<0.001 ^b^
	5	Discharge Medications ^c^	42 (10.9)	376 (97.2)	<0.001
	5	Medication Recommendations	148 (38.3)	378 (97.7)	<0.001
Other RCP Mandatory: Person completing summary	7	Grade ^c^	199 (51.6)	6 (1.6)	<0.001
	7	Specialty ^c^	76 (19.7)	1 (0.3)	<0.001 ^b^
	7	Date Discharge Record Completed ^c^	382 (99.0)	12 (3.1)	<0.001 ^b^
Non Mandatory	4	Discharge Method	83 (21.5)	131 (33.9)	<0.001
	5	Operations and Procedures	137 (35.5)	297 (76.7)	<0.001
	5	Allergies	5 (1.3)	140 (36.2)	<0.001 ^b^
	5	Risks and Warnings	52 (13.5)	135 (34.9)	<0.001
	5	Relevant Treatments and Changes made to Treatments	318 (82.4)	191 (49.4)	<0.001
	6	Hospital – Action	253 (65.5)	133 (34.4)	<0.001
	6	Hospital – Person Responsible	215 (55.7)	117 (30.2)	<0.001
	6	Hospital – Appropriate Data and Time	175 (45.3)	72 (18.6)	<0.001

^a^Chi squared unless otherwise stated

^b^Fishers exact test

^c^RCP mandatory item

## Discussion

Our study has demonstrated that the implementation of a NewEDS summary was associated with a 14.3% absolute increase in completeness of the RCP recommended handover information and a 15% absolute increase in the number of discharge summaries completed within 24 h of discharge.

Our finding also show a highly significant improvement in the quality of the discharge summaries being produced after the change in NewEDS. The likely mechanism for the improvements was, in part, due to the development of mandatory fields within the new electronic discharge summary which required the discharging doctor to input at least some information into specific parts of the form. However, this feature can still be bypassed by typing any characters such as “.” or “None” into the form and therefore does not ensure 100% completeness but has helped to make significant gains. Nevertheless, we recognise that there is still room for further improvement in the completeness of several of the data items.

We acknowledge that completeness of categories 6 and 7 (Table 2) including three RCP mandatory items relating to ‘person completing the summary’ (Table 3) showed a statistically significant decrease in the level of completeness after the implementation of NewEDS. This is because these items were not included as fields on the NewEDS system. This is why, for example, the inclusion of the ‘date that the discharge record was completed’ decreased from 99% to 3%. Furthermore the reduction in completeness for four non-mandatory items, was also probably because they were not included as explicit fields in the newEDS. Three of these related to hospital responsibility for future care of the patient and arguably these items should have been included in the NewEDS to give adequate information for primary care clinicians.

The improvements in timeliness may, in part, be attributed to the fact that the new electronic discharge summary is simpler to complete giving the increased number of automated fields where data is imported from the hospital database and thus does not require the clinician to input as much information manually. In addition there has been a strong focus within the NHS on timely completion of discharge summaries and an increased awareness of the need for discharge summaries to be completed within 24 h as a direct result of the introduction of the new electronic discharge summary [12].

It is worth noting that, despite the system change, some discharge summaries remained significantly delayed. In examining such cases individually it became apparent that there were two reasons for this: the first is that certain patients undergoing a complex period of care requiring multiple hospital attendances did not have clinical notes available at the time of discharge. The second may be attributed to periods of high intensity activity on the ward where these discharge summaries may be left to be completed in the next quieter period. In these cases, often concentrated at weekends and nights, it appears that clinical staff continued to prioritise other aspects of their work. This may also relate to relatively low staffing levels during these out-of-hours periods.

### Implications

Previous similar work that concentrated on the changes brought about by moving to an electronic discharge summary system from hand written communication was systematically reviewed by Motamedi et al. [14]. This showed a clear improvement in completeness of discharge summaries when electronic systems we used. Comparison of studies is, however, difficult as previous work does not have a definitive “gold standard” checklist to compare against. The advantage of this study is that we were able to use the carefully developed Royal College of Physicians checklist as an agreed standard [13].

Despite the fact that GPs have stated that they regard information about a patient’s medication as vitally important to facilitate appropriate onward care [2, 9, 15, 16] it has been noted by previous studies that this aspect of the discharge summary was often lacking. Kripalani et al. found that 21% to discharge summaries were missing discharge medications and Grimes et al. noted that the majority of failed medication reconciliation was due to missing information on discharge summaries. Kripalani et al. also showed significant increases in documentation of medication changes, discharge medications and medication recommendations when moving from a traditional to an electronic discharge summary. Our study would have demonstrated 100% completeness in all of these ‘medication’ categories had it not been for the fact that 12 of the summaries in the post implementation period were completed using the old forms. This occurred because in the early days of implementation some of the old forms were still available. We included these forms in our post implementation analysis as this reflects the reality that a small minority of these discharge summaries were not done using NewEDS.

Previous authors have demonstrated improvements and timeliness with the change to electronic discharge summary systems. Motamedi et al. showed 90.1% were sent out on or before the day of discharge but other authors including Kripalani et al. and Chen et al.[17], whilst demonstrating an improvement in timeliness with electronic systems, did not report this change in a comparable way.

This study demonstrates that it is possible to further refine and improve electronic discharge summaries addressing both completeness of information through the use of automated and required fields and also timeliness through a combination of easy completion of forms and a focus on the importance of this task. Furthermore, if these discharge summaries had been linked to Electronic Health Records (EHR) this would have provided opportunities for auto-importing of clinical information. This might have led to even more marked improvements in the completeness of the summaries.

This study demonstrates that both completeness and timeliness have increased when using the NewEDS.

### Strengths and limitations

This study has several strengths. Firstly it was conducted using a large sample size (773 summaries analysed in total) meaning the likelihood of the occurrence of a type II error is greatly reduced. This is important as much of the research conducted in this area has used only small sample sizes which impact on the reliability of the results. [2, 5, 15] In addition, the fact that data collection was spread over eight time points in a two year period demonstrated that the improvements brought about by the introduction of the new electronic discharge summary were sustainable and not just due to a short-term focus on a new system. Most importantly, we based the assessment of what information was deemed clinically important on a well validated piece of work carried out by the RCP. Although some items on this checklist may be viewed as subjective, we demonstrated a high level of agreement (99.23%) between two independent medical researchers when using this checklist to comparison sets of discharge summaries.

The major limitation of study methodology was that it was not possible to effectively blind the researchers and therefore it is possible that their reporting may have been subject to bias. The study design attempted to mitigate this risk by the use of two independent researchers with no involvement in the introduction of new electronic discharge summary system. There was also a potential for transcription error from the original paper collection to the Excel spreadsheet used in further analysis, however we calculated the data inputting error as 0.34% and therefore are confident that this would not have had an important influence on findings.

As our data collection defined only discharge summaries done on or before the day of discharge as timely, it is possible that we have under-estimated the number which were completed within the recommended 24 h period. A discharge summary done at 8 am for a patient discharged at 9 pm the previous day would not have been classed as timely in our study but would have met the recommendations. In addition, though most GP practices receive the discharge summaries via automatic e-mail with no time delay, some still need to be sent out by post and there was no way of measuring the delay brought about by this process or indeed the internal processes within GP practices which bring the discharge summaries to the attention of the relevant GP. In addition the study looked at implementation and effect on two busy, high turnover wards where the number of discharge summaries completed is very high. We have shown that the NewEDS was successful despite this service pressure but we have not formally tested other wards to demonstrate similar benefits.

Further limitations include the fact that we did not record data on the time needed to complete the discharge summaries; we did not include subjective assessments of the quality of discharge summaries by GPs who need to use them, and at least some of the improvement in the completeness score was driven by large changes in the rates of completion of administrative data such as GP practice code, gender, and method of admission.

Future work should be targeted at assessment of the accuracy of discharge summary information when compared to clinical notes and, importantly, to look for a link to an objective marker of improved patient outcomes such as reduction in avoidable readmissions.

## Conclusion

This research study shows that a structured, partially automated, electronic discharge system designed according to RCP standards improved the timeliness and completeness of discharge summaries. This should have a positive impact on safe transfer of care.

## Appendix

**Table 4:** A table showing the number of discharge summaries excluded, before reaching the desired sample size

		Overall number of DS	Overall number of DS in sample	Number of DS excluded			
				Missing	Unauthorised	Not discharged home	TOTAL excluded
B3	August 2010	179	50	3	1	4	8
	November 2010	158	50	21	0	6	27
	February 2011	213	50	6	1	5	12
	May 2011	177	50	4	0	2	6
	August 2011	188	50	3	0	0	3
	November 2011	215	50	2	0	0	2
	February 2012	127	50	0	0	7	7
	May 2012	214	50	0	1	2	3
D57	August 2010	61	50	1	8	1	10
	November 2010	63	43	1	8	10	19
	February 2011	57	43	1	8	5	14
	May 2011	59	50	1	5	2	8
	August 2011	76	50	1	0	1	2
	November 2011	51	48	0	1	2	3
	February 2012	52	47	1	0	3	4
	May 2012	47	42	1	0	4	5
